# Stimulated C2C12 Myotube Headspace Volatile Organic Compound Analysis

**DOI:** 10.3390/molecules29194527

**Published:** 2024-09-24

**Authors:** Tomos G. Rosser, Matthew A. Turner, James C. Reynolds, Neil R. W. Martin, Martin R. Lindley

**Affiliations:** 1 School of Sport Exercise and Health Sciences, Loughborough University, Loughborough LE11 3TU, UKn.r.w.martin@lboro.ac.uk (N.R.W.M.); 2 Department of Chemistry, School of Sciences, Loughborough University, Loughborough LE11 3TU, UK; m.a.turner@lboro.ac.uk (M.A.T.); j.c.reynolds@lboro.ac.uk (J.C.R.); 3 School of Health Sciences, University of New South Wales, Sydney 2050, Australia

**Keywords:** VOC, EPS, skeletal muscle, gas chromatography, mass spectrometry

## Abstract

Understanding exercise metabolism and the relationship with volatile organic compounds (VOCs) holds potential in both health care and sports performance. Exercise metabolism can be investigated using whole body exercise testing (*in vivo*) or through the culture and subsequent electrical pulse stimulation (EPS) of myotubes (*in vitro*). This research investigates the novel headspace (HS) analysis of EPS skeletal muscle myotubes. An *in vitro* system was built to investigate the effect of EPS on the volatile constituents in the HS above EPS skeletal muscle. The C2C12 immortalised cell line was chosen. EPS was applied to the system to induce myotube contraction. The *in vitro* system was applied to the analysis of VOCs using thermal desorption (TD) sampling. Samples were collected under four conditions: environmental samples (enviro), acellular media HS samples (blank), skeletal muscle myotubes without stimulation HS samples (baseline) and EPS of skeletal muscle myotube HS samples (stim). TD sampling combined with gas-chromatography mass spectrometry (GC-MS) detected two compounds that, after multivariate and univariate statistical analysis, were identified as changing due to EPS (*p* < 0.05). These compounds were tentatively assigned as 1,4-Dioxane-2,5-dione, 3,6-dimethyl- and 1-pentene. The former is a known lactide and the latter has been reported as a marker of oxidative stress. Further research should focus on improvements to the EPS system, including the use of more relevant cell lines, quantification of myotube contractions, and the application of targeted analysis, metabolic assays and media analysis.

## 1. Introduction

The potential of volatile organic compounds (VOCs) to describe exercise metabolism is yet to be unharnessed but holds significant benefit for health applications and investigating exercise performance. Exercise metabolism can be investigated using whole body exercise testing (*in vivo*) or through the culture and subsequent electrical pulse stimulation (EPS) of myotubes (*in vitro*).

*In vitro* models have been designed to simulate biological responses to stimuli for decades [1,2]. From gene expression [3] to protein uptake [4], cancer research [5] and drug discovery [6], cell models have provided information on molecular mechanisms that has enhanced biological understanding. 

As metabolic function is driven by skeletal muscle activity and thus exercise, *in vitro* models applying EPS to induce biomarker expression representative of exercise hold great potential. 

Cultured C2C12 murine skeletal muscle cells are commonly used in experiments to study responses that convey *in vivo* physiological responses in the human biological system. EPS-induced contraction of cultured C2C12 myotubes has been reported to exhibit similar responses in both gene and protein expression [7], glucose uptake [8,9], electrochemical gradients [10] and muscle hypertrophy [11] to the exercise-mediated physiological effects *in vivo*. Investigations using *in vitro* models can provide a proof of concept regarding unexplored *in vivo* physiological responses. A novel concept can be explored initially *in vitro* and then explored further *in vivo* in the pursuit of understanding the mechanisms that underpin health and disease.

VOCs have been used to differentiate between skeletal muscle of different animal species, including human tissue [12,13] and between four different pork muscle groups [14]. All samples presented in the studies were obtained postmortem. Currently, only one study has reported headspace (HS) analysis of VOCs produced or consumed by skeletal muscle *in vitro*, reporting VOCs from the HS of rat L6 skeletal muscle cells [15]. The researchers aimed to identify and quantify VOCs produced or consumed by differentiated skeletal muscle using needle trap extraction (NTE) gas-chromatography mass spectrometry (GC-MS). Sixteen species of volatile organic compounds were identified as products of skeletal muscle metabolism and seven were identified as substrates of skeletal muscle metabolism. Of the products, aldehydes were the prevailing species, with two esters and dimethyl disulphide (DMDS) also detected. It was proposed that the presence of aldehydes was related to either aldehyde dehydrogenase (ALDH) converted to carboxylic acids or alcohol dehydrogenases converted to alcohols. The author leaned towards ALDH activity being the probable explanation due to substrate availability. The two esters were linked with carboxylesterase (CES) activity and the hydrolysis of esters into carboxylic acids and alcohols. Little was mentioned about the disulphide compound. However, further research has suggested that DMDS possesses antimicrobial properties and could be active in the prevention of bacterial growth within the culture [16].

In the same study, the seven compounds identified as substrates of skeletal muscle metabolism consisted of two ketones; three hydrocarbons, including isoprene and pentane; and two sulphur compounds, including dimethylsulfide [15]. The ketone production has been associated with the oxidation of secondary alcohols catalysed by ADH [17] or β-oxidation of branched chain fatty acids (BCFA) and the presence of dimethyl sulphide as a potential product of the sulphur-containing amino acids methionine and cysteine in the transamination pathway [18]. 

GC-MS is the dominant analytical technique deployed in VOC analysis with solid phase micro-extraction (SPME), GC-MS is the dominant analytical technique deployed in VOC analysis with solid phase micro-extraction (SPME) & TD the common sample collection methods [19], with needle trap extraction (NTE) sampling also reported [15]. Other powerful techniques for analysing VOCs include ion mobility mass spectrometry (IMS), proton transfer reaction mass spectrometry (PTR-MS), selective ion flow tube mass spectrometry (SIFT-MS), secondary electrospray mass spectrometry (SESI-MS) and atmospheric pressure chemical ionisation (APCI). IMS has been applied to VOC skin analysis [20], while PTR-MS analysis of urine VOCs has been reported [21]. PTR-MS, SIFT-MS, SESI-MS and APCI have all reported applications in real time breath analysis, holding potential in the field of in vivo exercise metabolism VOC analysis [22,23,24,25].

Electrical pulse stimulation (EPS) of skeletal muscle cells represents an *in vitro* exercise model [26], and headspace (HS) analysis of volatile organic compounds (VOCs) provides a technique for the investigation of exercise-mediated metabolites. The detection of exercise-mediated VOCs will enhance the application of EPS of skeletal muscle, offering a novel method of metabolic measurement.

The research presented investigated changes in the VOC profile of skeletal muscle myotubes following electrical pulse stimulation. EPS was applied to terminally differentiated C2C12 skeletal muscle myotubes for 12 h. Automated headspace sampling captured VOCs on thermal desorption tubes from comparable environments. Thermal desorption samples were analysed using gas chromatography mass spectrometry

## 2. Results

An *in vitro* system capable of inducing C2C12 skeletal muscle myotube contraction through EPS was successfully applied to the sampling of headspace VOCs. Through Mass Hunter Quantification software (version 12.1), a range of organic compounds were detected, including alcohols, carboxylic acids, ketones, aldehydes, hydrocarbons and cyclic hydrocarbons. To investigate any discriminating compounds between samples collected before stimulation and after twelve hours of stimulation, supervised principal component analysis (PCA) and unsupervised orthogonal partial least square-discriminative analysis (OPLS-DA) was applied. Nine compounds were found to increase/decrease before and after 12 h of EPS (Table 1). Three compounds decreased and six compounds increased, all identified using National Institute of Science and Technology Mass Spectral Library (NIST 17). 

Thermal desorption gas chromatography mass spectrometry (TD-GC-MS) analysis of the HS above stimulated skeletal muscle myotubes successfully provided a sampling method with sufficient sensitivity to detect changes in metabolites mediated by EPS. 

A non-significant change (*p* > 0.5) in the response of both Ethanol and Acetic acid was observed after twelve hours of EPS, with both compounds displaying high variability in the responses detected in the samples.

Of the remaining seven compounds, n-Hexadecanoic acid and Benzene displayed non-significant increases (*p* > 0.05) after twelve hours of EPS. Octadecane and Hexadecane both displayed non-significant decreases (*p* > 0.5). Significant changes (*p* < 0.05) were observed in the environmental samples for Octadecane and the blank samples for Hexadecane, indicating the source was not from the EPS of C2C12 myotubes. 

**Figure 1:**
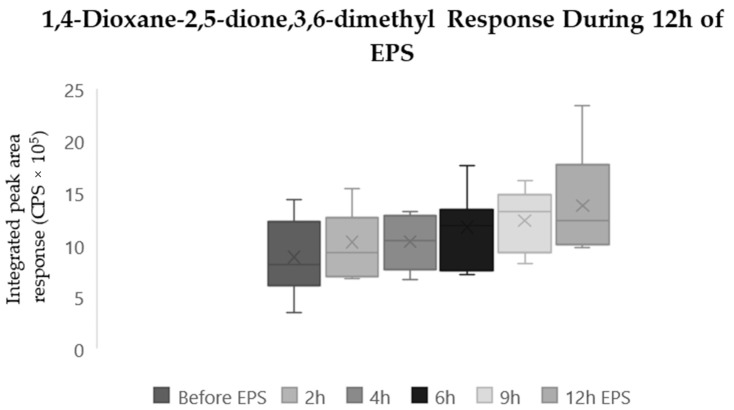
Box and whisker plot of 1,4-Dioxane-2,5-dione, 3,6-dimethyl response during twelve-hour EPS experiments. N = 6.

A related samples Wilcoxon rank test identified significant increases (*p* < 0.05) after twelve hours of EPS in the responses of the remaining three compounds: Benzoic acid, 1-Pentene, and 1,4-Dioxane-2,5-dione, 3,6-dimethyl. A related samples Friedmans two-way analysis of the variance by ranks found no significant differences in the distribution of the Benzoic acid response (*p* > 0.2). Significant increases (*p* < 0.01) we observed in 1,4-Dioxane-2,5-dione, 3,6-dimethyl between samples collected before and after twelve hours of EPS (Figure 1). A related samples Friedmans two-way analysis of the variance by ranks found significant differences in the distribution of the 1,4-Dioxane-2,5-dione, 3,6-dimethyl response (*p* < 0.001). Box and whisker plots of the 1,4-Dioxane-2,5-dione, 3,6-dimethyl response, detected in samples collected at the six sampling points, display a mean increase of 60% from rest (before EPS) after nine hours of EPS, followed by a plateau after twelve hours of EPS (Figure 1). No significant changes were observed in any of the other sample conditions (Base, Blank, Media, Enviro). The compound identified as 1,4-Dioxane-2,5-dione, 3,6-dimethyl, also known as Lactide, is the dimer of lactic acid. The steady increase during the stimulation may indicate that the cellular metabolism was anaerobic in nature.

**Figure 2:**
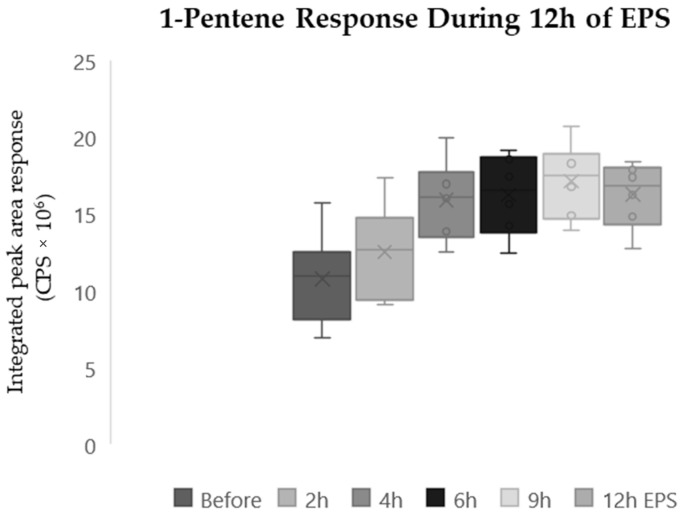
Box and whisker plot of 1-Pentene response during twelve-hour EPS experiments. N = 6. *p* < 0.001.

Finally, 1-Pentene also increased significantly (*p* < 0.05) after twelve hours of EPS (Figure 2). A mean increase of 79% was observed before EPS and in the responses after twelve hours of EPS. A related samples Friedmans two-way analysis of the variance by ranks found significant differences in the distribution of the 1-Pentene response (*p* < 0.001). Box and whisker plots of the 1-Pentene response detected in samples collected at the six sampling points display an increase after four hours of EPS (mean increase 50%) followed by a plateau after six hours of EPS (Figure 2). Two compounds demonstrated significant changes during stimulation that were not observed in any controlled conditions. 1,4-Dioxane-2,5-dione, 3,6-dimethyl (Figure 1) and 1-pentene (Figure 2) were both observed to significantly increase (*p* < 0.05) during 12 h of EPS. 

## 3. Discussion

Electrical pulse stimulation of skeletal muscle myotubes was used to investigate exercise-mediated physiology *in vitro*. The volatile organic compound composition was shown to reflect the metabolic state *in vivo*. Analysis of headspace VOCs presents an opportunity to explore skeletal muscle-specific volatiles consumed and produced in a simulated exercise environment.

The research presented set out to investigate changes in the VOC profile of skeletal muscle myotubes following electrical pulse stimulation. SIMCA analysis identified eleven compounds that changed during 12 h of stimulation. Two of these compounds were found to be significantly increased in simulated environment compared to the controls.

The two compounds were identified as significantly changed in the samples collected after twelve hours of electrical pulse stimulation (EPS) when compared to samples collected before EPS. The detected compounds were identified in the National Institute of Science and Technology Mass Spectral Library (NIST 17) as 1,4-Dioxane-2,5-dione 3,6-dimethyl and 1-Pentene.

1,4-Dioxane-2,5-dione, 3,6-dimethyl is a known lactide. Lactide can form from the cyclical esterification of two lactic acid molecules [27]. Whether the source of lactide is lactic acid is open to debate, and the detection of significant increases in the response of lactide during an *in vitro* model of exercise promotes speculation as to its origin. 

In this respect, it would have been of interest to conduct a lactic acid assay of the cellular medium after twelve hours of EPS. An increase in lactide formation during twelve hours of stimulation may represent the anaerobic metabolism of skeletal muscle and the formation of lactate as a by-product. Should this relationship be demonstrated, it could provide validity to this cell model as able to induce exercise-specific responses in skeletal muscle. Measuring C2C12 lactate production through the measurement of gene expression would confirm this [28].

Similarly, the compound identified as 1-Pentene has metabolic significance. Hydrocarbons have been reported as markers of oxidative stress in the breath [29,30]. Hydrocarbons, including alkenes, have been identified as a marker of oxidative stress [19,30,31] and could be reflecting the creation of an oxidative stress environment because of the electrical pulse stimulation of the myotubes.

It is well established that exercise induces oxidative stress in skeletal muscle. The formation of reactive oxygen species (ROS) is both beneficial and detrimental to the function of muscle [32]. The production of an oxidative stress marker is a promising result that supports the use of *in vitro* cell EPS research as a model of exercise. Liquid chromatography mass spectrometry (LC-MS) of the cell medium would confirm the presence of any ROS [33].

Understanding the origins and relevance of VOCs will enhance areas of research, including breath research and non-invasive clinical practices. Understanding exercise-specific VOCs will aid in the discovery of non-invasive markers of metabolism, health and fitness. HS VOC analysis of skeletal muscle *in vitro* will provide a platform for targeted analysis of exercise-regulated compounds and investigations in integrated human biology.

While EPS has become more prevalent in skeletal muscle research, the application of VOC analysis to EPS has not been seen previously. This paper investigated the novel concept that electrical pulse stimulation (EPS) changes the volatile organic compound (VOC) composition of the headspace above skeletal muscle myotubes. It is the first known report of VOC analysis of the headspace of stimulated C2C12 myotubes. The paper describes the design of a novel system that combines the comprehensively researched areas of VOC headspace analysis and EPS of C2C12 myotubes.

The application of this novel collaboration of systems has produced the first reported set of results attributing VOC HS changes to EPS of C2C12 myotubes. Two compounds were identified to have increased in the stimulated cell environment. The EPS C2C12 myotube system has the potential to provide rudimentary insight into skeletal muscle metabolism during exercise. Analysis of the VOCs produced in this system provides a proof of concept showing that VOC composition changes with the application of EPS.

The implications of this research transcend cell culture and pose a potential understanding of the integrative biological mechanisms occurring during exercise metabolism. The future development of a system that better represents human skeletal muscle metabolism holds the potential to comprehensively investigate the effect of skeletal muscle contraction on endogenous VOC concentrations both *in vitro* and *in vivo*.

Limitations were identified in the study. The stainless steel sample line, transporting HS VOCs from the incubator to the multisampler, could potentially cause condensation and therefore loss of volatiles. A heated sample line would mitigate any losses in VOCs in future studies. Additionally, evidence of myotube contractile activity was exclusively qualitative. The inclusion of quantitative functional and morphological readouts and myotube analysis would enhance the contraction criteria, including any effect contraction coverage or myotube size has on VOC composition. Finally, no internal standards were used. 

Future *in vitro* investigations will lead to the understanding of skeletal muscle-specific VOCs. This will lend itself to collaboration with other biological systems e.g. liver or lung, to investigate the effect of alternative biological tissue on VOC concentrations. Furthermore, the *in vitro* system could be used to investigate *in vivo* VOC concentrations related to exercise metabolism. VOC breath analysis in sport and exercise metabolism will benefit from an understanding of skeletal muscle VOCs identified in an isolated environment. Understanding exercise-induced VOCs in the breath compendium will produce a greater knowledge and understanding of the 1500 breath components. This in turn will lead to a greater understanding of VOCs related to disease states and health. This knowledge will aid in unlocking the potential of breath analysis as a non-invasive measurement of health and wellbeing.

An improvement in the analysis of HS volatiles from stimulated skeletal muscle myotubes would be the application of dynamic HS analysis. The current research operated a static HS analysis within a partially closed system. Without a completely closed system, there is an increased opportunity for volatiles to transfer out of the HS and into the environment. Dynamic HS analysis is a potential technique to mitigate this limitation [15,34].

## 4. Materials and Methods

### 4.1. Cell Culture and Differentiation

**Figure 3:**
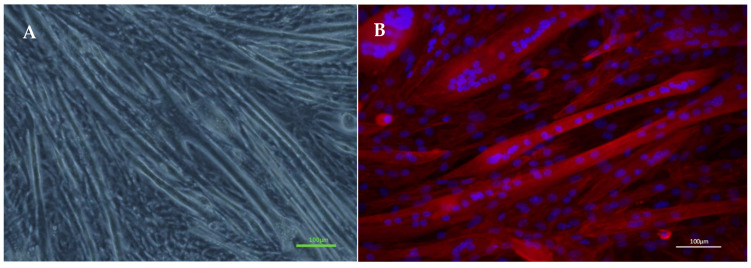
(**A**) Photo of C2C12 myotubes following nine days in differentiation medium. (**B**) Fluorescence image of myotubes stained with DAPI and RP. The images presented are an overlay of the DAPI staining (cell nuclei—blue) and the rhodamine phalloidin (actin filaments—red) staining images. Scale bar indicates 100 µm. Microscope: LEICA, DMIL LED, HI PLAN|10×/0.22.

C2C12 murine skeletal muscle myoblasts (RRID: CVCL_0188; ECACC, Sigma Aldrich, Dorset, UK) were used for all experimentation and cultured in T75 flasks (Nunc™, Thermo Fisher Scientific, Leicestershire, UK) maintained in a humidified 5% CO_2_ incubator (HERAcell 240i, Thermo Fisher, Leicestershire, UK). Cells were maintained in growth media consisting of high glucose Dulbecco’s Modified Eagle’s Medium (DMEM, Sigma-Aldrich, Dorset, UK), 20% foetal bovine serum (FBS, Dutscher Scientific, Hessle, UK) and 1% penicillin streptomycin (Thermo Fisher Scientific, Leicestershire, UK) until 80% confluent, at which point the cells were enzymatically detached from the flasks with 1 mL of trypsin enzyme (Trypsin-EDTA solution, Sigma Aldrich, Dorset, UK). Cells were counted using the trypan blue exclusion method. C2C12 myoblasts were seeded into six-well cell culture plates (Nunc™ Delta Surface, Thermo Scientific, Leicestershire, UK) at a density of 1 × 10^5^ cell/well in 2 mL of growth medium and incubated. Once the seeded myoblasts reached 80% confluence, differentiation was induced using a differentiation medium: high glucose DMEM, 2% horse serum (Sigma–Aldrich, Dorset, UK), and 1% penicillin streptomycin and incubated. The differentiation medium was changed every 3 days. After nine days in differentiation medium, the myotubes were prepared for electrical pulse stimulation (Figure 3A). Fluorescence imaging was used to demonstrate multi-nucleic structure of differentiated myotubes. Cells were fixed with formaldehyde and the myotubes were stained with 4′,6-diamidino-2-phenylindole (DAPI, 0.1%) and rhodamine phalloidin (RP, 0.5%) in tris buffered saline (TBS). During the staining process, the RP and DAPI bound to the actin filaments and cell nuclei, respectively, and when exposed to fluorescent light, absorbed specific wavelengths (Figure 3B).

### 4.2. EPS

**Figure 4:**
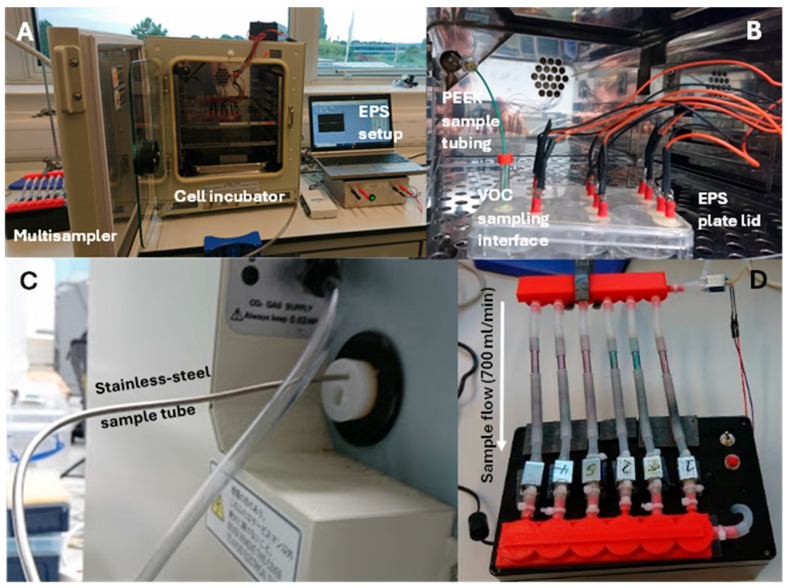
Laboratory set-up for thermal desorption sampling of the headspace of stimulated C2C12 myotubes. (**A**) Open view of the experimental set up with the multisampler on the left, incubator in the middle and the EPS kit setup to the right. (**B**) A view inside the incubator of the EPS plate sitting on the top shelf with the PEEK tubing fitted to the VOC sampling interface (clear glass centrifuge vial (12 mm diameter) fitted with a T/S/T septa). (**C**) Stainless steel sampling tube enters/exits the incubator through a probe hole and is connected to the PEEK tubing inside the incubator. (**D**) Remote multi-sampler connected to the stainless steel sample tubing from the incubator to the six TD tubes. The arrow depicts the direction of sample flow (700 mL/min) through the TD tubes.

The EPS plate lid, consisting of 6 pairs of electrodes, was sterilised and fitted onto the six-well plate that contained the differentiated skeletal muscle myotubes. This was completed in a biological cabinet (Figure 4A) and then the cells were incubated overnight before experimentation. An electrical current was generated (National Instruments stimulus generator) and introduced to the cell medium to induce myotube contraction. An EPS output (20 V) was applied for 12 h.

### 4.3. TD Sampling

To facilitate HS sampling from the EPS kit, a sampling interface was developed. A clear glass centrifuge vial (12 mm diameter) fitted with a T/S/T septa and with the end cut off was epoxied into a pre-drilled hole in the electrode head plate to allow access to the HS above the cell culture media. 

To facilitate thermal desorption (TD) analysis, an in-house bespoke remote multi-sampler was developed to have the capacity automated sampling of HS VOCs into TD tubes. The remote multi-sampler consisted of a vacuum pump (Karlsson Robotics, D202813, 12 VDC, Tequesta, FL, USA), 12 V power pack (Power Buck module DC-DC stepdown power supply, JZK24/12V–5V 5A), Arduino nano bluetooth module (DAOKAI, HC-06), six two-way solenoids and two 3D-printed TD tube manifolds. Each TD tube was fitted to both manifolds using silicon tubing (Figure 4D). The multisampler vacuum pump pulled a sample flow (700 mL/min) from the incubator via polyetheretherketone (PEEK, Thermo Fisher Scientific, Leicestershire, UK) tubing and stainless steel tubing to the TD tubes. PEEK tubing pierced the T/S/T septa on the EPS sampling interface to allow access to the HS (Figure 4B). The sample line exited out of the incubator through a sealed probe hole (Figure 4C), transporting the sample to the TD tubes. The programmed two-way solenoids allowed the vacuum pump to pull the sample flow through a single TD tube whilst the others remained switched off. 

During the stimulation period, VOCs were extracted from the HS of the six-well plate and preconcentrated in a TD tube (Biomonitoring inert coated stainless steel–Markes International Ltd., Bridgend, UK) before analysis by GC-MS.

The cell culture HS was sampled for one minute in a TD tube at a flow of 700 mL/min. Following the experimental run, each TD tube was capped, sealed and kept in the refrigerator (4 °C) until analysis. During each experimental run, an HS-TD sample was taken immediately before (pre) and after 2, 4, 6, 9 and 12 h of EPS from the same batch of cells using the remote multisampler.

The Markes Unity-xr Thermal Desorber (Markes International Ltd., Bridgend, UK) was operated using Markes Instrument Control (version 1.0.0). Sample tubes were desorbed under a flow of helium (50 mL/min) at a temperature of 300 °C for five minutes. The resultant VOC plume was subjected to cryofocussing at −10 °C using a Peltier-cooled cold trap (U-T2GPH-2S Focusing trap, General Purpose (Hydrophobic), Markes International Ltd., Bridgend, UK). The cold trap was then desorbed at 300 °C for five minutes to inject volatiles into the GC-MS system. The temperature of the transfer line from the thermal desorber to the GC (2 m × 0.25 µm, deactivated non-polar fused silica column, part number 64,339, Markes International Ltd., Bridgend, UK) was set to 200 °C, and a heated valve was also set at 200 °C. Table 2 describes the Markes International Unity-xr thermal desorption parameters used throughout the study.

The EPS system with TD sampling was applied to detect and measure the VOCs formed during stimulation of C2C12 skeletal muscle myotubes. HS samples were collected under five conditions, Stim, Base, Blank, Media and Enviro (Table 3). 

### 4.4. GC-MS

GC-MS analysis, using electron ionisation (EI), was performed using an Agilent Technologies 7820A GC system fitted with a non-polar Agilent HP-5MS 5% Phenyl Methyl Siloxane 30 m × 0.25 mm × 0.25 µm GC column coupled to an Agilent Technologies 5977B MSD single quadrupole mass analyser (Agilent Technologies LDA UK Ltd., Didcot, UK). The GC-MS systems were operated using MSD Chemstation Data Analysis Mass Hunter GC/MS Acquisition (version: B.07.06.2704 Agilent Technologies LDA UK Ltd., Didcot, UK).

The GC oven method (Table 3) began with an initial temperature 40 °C with a two-minute hold, ramped up to 250 °C at 10 °C/min and was held for one minute. The helium carrier gas was set at a flow rate of 2.0 mL/min. The MS parameters were as follows: full scan mode, *m*/*z* range 40–500 at 5.6 scan/s, with no solvent delay. The ion source temperature was 230 °C, the MS quad temp was 150 °C, and the mass transfer line temperature was 300 °C.

### 4.5. GC-MS Data Processing

A daily retention index of the standard was used. A targeted Agilent MassHunter Quantitative (Version: B.09.00, Agilent Technologies LDA UK Ltd., Didcot, UK) method for each retention index sample was developed and applied to each daily retention index run. The extracted retention times (RT) for eight compounds with known retention indices (RI) were used to generate retention time calibration (RTC) files for each day of analysis. Each RTC file was applied to the library search method for samples analysed on the corresponding day of analysis. Compound assessment and identification was completed using Agilent MassHunter Quantitative (Version: B.09.00 Agilent Technologies LDA UK Ltd., Didcot, UK).

### 4.6. Statistical Analysis

The excel data matrix of integrated responses was imported into SIMCA Multivariate Statistics Analysis Software (version 17, Sartorius, Göttingen, Germany). Discriminating compounds were identified and imported into IBM SPSS Statistics (version 25, New York, NY, USA). Non-parametric tests were applied to determine the significance of the variance between samples. A Wilcoxon rank test was applied to two classes to determine whether there was a significant difference in the variation.

Variables that elicited significant changes were investigated further using a related samples Friedmans two-way analysis of the variance to determine the significance of the changes across the time points of all samples. A significance level of 95% (*p* < 0.005) was applied during this analysis.

## 5. Conclusions

Increased volatile organic compounds (VOCs) during electrical pulse stimulation (EPS) was identified using thermal desorption gas chromatography (TD-GC-MS). Although compound identification of both 1-pentene and 1,4-Dioxane-2,5-dione, 3,6-dimethyl need confirmation, the production of two compounds during twelve hours of EPS was detected. 

To progress this work, the following steps would be of interest:

1. Confirmation of the identity of detected biomarkers by comparison with spectra and RI of authentic standards.
2. Collection of additional replicate data to investigate the variability of the compounds detected and explore any correlations between significant biomarkers.
3. Targeted work on biomarkers. Targeted analysis will increase the sensitivity and lead to improved quantification of targeted metabolites under different conditions. This will promote the application of such a model in the in vitro analysis of exercising muscle.

This is the first report of VOC data collected from the HS of stimulated C2C12 myotubes. The identification of exercise-specific markers in a cell model holds promise in understanding health and disease.

Further research should focus on improvements to the EPS system, including the use of more relevant cell lines and quantification of myotube contraction.

## Figures and Tables

**Table 1:** Discriminating compounds from OPLS-DA model of TD-GC-MS samples collected before and after twelve hours of EPS. A compound’s tendency to increase or decrease, as observed from the OPLS-DA model, has been described. ↑, Increase. ↓, Decrease. National Institute of Science and Technology Mass Spectral Library (NIST 17) match and reverse and reverse match scores are detailed.

NIST (17) Match	Prob Match %	Match	R. Match	Increase/Decrease
Ethanol	89.0	947	951	↓
1-Pentene	23.9	787	871	↑
Acetic Acid	89.0	866	889	↑
Benzene	68.2	677	955	↑
Benzoic acid	20.7	814	846	↑
1,4-Dioxane-2,5-dione, 3,6-dimethyl	70.4	637	909	↑
Hexadecane	29.3	954	961	↓
Octadecane	22.2	805	907	↓
-n-Hexadecanoic acid	63.4	844	893	↑

**Table 2:** Experimental sample definitions.

Reference	Sample Type	Description
Stim	Stimulated samples	Electrical pulse-stimulated C2C12 murine skeletal muscle myotubes
Base	Baseline samples	Non-stimulated C2C12 murine skeletal muscle myotubes
Blank	Blank samples	Electrical pulse-stimulated cell culture differentiation media
Media	Media samples	Non-stimulated cell culture differentiation media
Enviro	Environmental samples	Cell culture incubator

**Table 3:** TD-GC-MS instrumentation operating parameters.

Parameter	Level
Thermal Desorption	
Sample Time	1 min
Tube Temperature	300 °C
Tube Desorption	5 min
Trap Temperature (low)	−10 °C
Trap Temperature (high)	300 °C
Trap Desorption	5 min
Trap Flow	50 mL/min
Split flow	Splitless injection
Gas Chromatography	
Column dimensions	30 m × 0.25 mm × 0.25 μm
Column phase	HP-5MS 5% Phenyl Methyl Siloxane
Carrier gas	Helium
Column flow	2.0 mL/min
Total run time	29 min
Temperature Program	
40 °C	2 min hold, 10 °C/min
300 °C	1 min hold
Mass Spectrometry Electron Ionisation	
Mass Range	*m*/*z* 40–*m*/*z* 500
Solvent delay	0.1 min
Ionisation time	0.1 min
Scan time	5.9 scan/s
Transfer line temperature	300 °C
Ion source temperature	230 °C
MS quad temperature	150 °C

## Data Availability

The datasets presented in this article are not readily available because the data are part of an ongoing study. Requests to access the datasets should be directed to the corresponding author m.lindley@unsw.edu.au.
